# A New DGNSS Positioning Infrastructure for Android Smartphones

**DOI:** 10.3390/s20020487

**Published:** 2020-01-15

**Authors:** Duojie Weng, Xingli Gan, Wu Chen, Shengyue Ji, Yangwei Lu

**Affiliations:** 1 Department of Land Surveying and Geo-Informatics, The Hong Kong Polytechnic University, Hong Kong M1504, China; wengduojie@aliyun.com (D.W.); wu.chen@polyu.edu.hk (W.C.); lu.y.lu@polyu.edu.hk (Y.L.); 2 State Key Laboratory of Satellite Navigation System and Equipment Technology, Shijiazhuang 050081, China; 3 The 54th Research Institute of China Electronics Technology Group Corporation, Shijiazhuang 050081, China; 4 Department of Science and technology of Surveying and Mapping, China University of Petroleum (East China), Qingdao 266000, China; jidifferent@gmail.com

**Keywords:** GNSS, DGNSS, smartphone, android, infrastructure, accuracy

## Abstract

One’s position has become an important piece of information for our everyday lives in a smart city. Currently, a position can be obtained easily using smartphones that is equipped with low-cost Global Navigation Satellite System (GNSS) chipsets with accuracy varying from 5 m to 10 m. Differential GNSS (DGNSS) is an efficient technology that removes the majority of GNSS errors with the aid of reference stations installed at known locations. The sub-meter accuracy can be achieved when applying the DGNSS technology on the advanced receivers. In 2016, Android has opened the accesses of raw GNSS measurements to developers. However, most of the mid and low-end smartphones only provide the data using the National Marine Electronics Association (NMEA) protocol. They do not provide the raw measurements, and thus do not support the DGNSS operation either. We proposed a DGNSS infrastructure that correct the standalone GNSS position of smartphones using the corrections from the reference station. In the infrastructure, the position correction is generated considering the GNSS satellite IDs that contribute to the standalone solution in smartphones, and the position obtained is equivalent to the solution of using the range-domain correction directly. To serve a large number of smartphone users, a Client/Server architecture is developed to cope with a mass of DGNSS positioning requests efficiently. The comparison of the proposed infrastructure against the ground truth, for all field tests in open areas, showed that the infrastructure achieves the horizontal positioning accuracy better than 2 m. The improvement in accuracy can reach more than 50% for the test in the afternoon. The infrastructure brings benefits to applications that require more accuracy without requiring any hardware modifications.

## 1. Introduction

Global Navigation Satellite System (GNSS) measures the satellite-to-user distances to determine the user position, suffering from measurement errors caused by the satellite clock, the satellite orbit, the propagation delay, and the receiver. The standalone GNSS determines position with an accuracy of 5 m to 10 m, and brings many benefits to our daily life [1]. Some applications, however, such as the lane-level positioning system and the collision warning system, and the Geographic Information System (GIS) information collection system, require far better accuracy than that given by the standalone GNSS [2].

The concept of Differential GNSS (DGNSS) was developed in the 1980s. DGNSS reduces measurement errors using corrections or raw measurements generated from one or more reference stations [3,4]. The GNSS errors are correlated over space, and the corrections from reference stations remove all of the satellite clock errors, most of the orbit errors, and most of the ionospheric and tropospheric errors on the user side. The positioning accuracy of the DGNSS user can be improved up to 1 m to 2 m, which is sufficient for many applications. In intelligent transportation systems, DGNSS is integrated with other sensors to enhance accuracy and reliability, and, in this case, the installation of a special DGNSS unit has been required in such vehicle control and safety systems [5,6,7,8,9]. The DGNSS position error grows with the distance between the reference station and the user receiver. The deterioration with distance is mainly caused by the de-correlated error of ionosphere, which can be mitigated using the ionospheric gradients [9,10]. Note that the receiver-related error (e.g., multipath errors) could not be reduced using corrections. In previous studies, the multipath errors can be mitigated with the aid of inertial navigation system/computer vision [5,11], cooperative positioning [6,7], and a three-dimensional map [8].

In recent years, a significant change in GNSS is that most smartphones are equipped with consumer-grade GNSS chipsets. According to the Global System for the Mobile Communication Assembly (GSMA) report, the smartphone market would reach at least 2.9 billion in 2020 [12]. DGNSS is disabled in the chipsets embedded within most smartphones in the electronic market, that is, DGNSS could not be achieved by feeding range corrections into the GNSS chipsets, as is achieved in the conventional DGNSS units. In 2016, Google has opened the access to raw GNSS measurements [13]. This has brought great benefits to applications that require higher accuracy. With the newest smartphones, the centimeter positioning accuracy can be achieved by using precise point positioning (PPP) or real-time kinematic positioning (RTK) [14,15]. However, most of the smartphones do not provide continuous carrier phase measurements due to the great power consumption for normal smartphones. In addition, most of the mid and low-end smartphones do not provide the pseudorange measurements, and thus do not support the DGNSS operation.

At the early stage of development of GNSS, the position domain correction was developed to improve the GNSS accuracy. This simple method assumes that the same set of satellites is utilized to determine the positions of the reference station and the smartphones. However, buildings or trees on the path from the satellite to the user can block the GNSS signals, and the reference station and users would utilize different sets of satellites. Thus, the conventional position correction degrades the accuracy of GNSS most of the time, instead of improving the accuracy [16,17,18].

The concept of DGNSS based on National Marine Electronics Association (NMEA) messages was first proposed in [17], and subsequently demonstrated in [18,19]. These research efforts have shown that the positioning accuracy of smartphones can be improved in mid-latitude regions, and have demonstrated the feasibility of the concept. The line-of-sight of each satellites should be computed in the new concept. In [17], a method of computing the line of sights based on NMEA sentences was also presented.

In recent years, smartphones have become ubiquitous, and the accuracy requirement for smartphones has become more demanding in applications as diverse as agriculture, transport, and social apps. In this paper, we present details of the DGNSS technology for smartphones from which raw GNSS measurements are not accessible. The computation burden of the technology is significantly large for smartphones. We proposed a DGNSS infrastructure that corrects the standalone position from smartphones using the corrections from the reference station. A Client/Server architecture is presented to cope with a mass of DGNSS positioning requests efficiently. Here, we describe the derivation of the DGNSS based on NMEA messages and show how to solve the technical problems when implementing the infrastructure. Specifically, we address issues such as the fundamentals of the DGNSS based on NMEA messages, the essential assumptions made on the smartphone GNSS chipsets, and the infrastructure implementation issues. Different experiments are carried out to assess the performance of the infrastructure considering different effects, including the ionospheric effects, the multipath effects, and different smartphone types.

## 2. Background

One of the basic observables available in the GNSS receiver is the pseudorange (ρ), which contains the true range r contaminated by various errors:

(1) ρ = r + b − B + I + T + M + e,

where b and B denote the receiver and satellite clock error; I and T are the ionospheric delay and the tropospheric delay, respectively; M represents the multipath error; and e represents the error caused by receiver noise and interferences. Note that the pseudorange in Equation (1) contains other errors, such as the relativistic error and the differential code bias. For simplicity consideration, these terms are not included.

Most of the errors in Equation (1) can be reduced either using navigation messages broadcast from satellites, or applying pseudorange corrections from a nearby reference station. These two approaches are known as the standalone GNSS and the range domain DGNSS. This section presents the fundamentals, and then introduces the NMEA protocol, which is widely employed to deliver positioning information to users in most of the GNSS chipsets.

### 2.1. Standalone GNSS

The standalone GNSS employs the navigation messages to correct some errors in Equation (1):

(2) $$\rho_{c}=\rho -\hat{I}-\hat{T}+\hat{B},$$

where $\rho_{c}$ denotes the corrected pseudorange; $\hat{I}$ and $\hat{T}$ are the estimates of the ionospheric delay and the tropospheric delay, respectively; and $\hat{B}$ is an estimate of the satellite clock error. For the single frequency receiver, $\hat{I}$ is estimated using various models. One of the examples is the Klobuchar model that removes approximately 50 percent Root Mean Square (RMS) of ionospheric errors, requiring only eight parameters as a part of navigation messages [20]. Furthermore, $\hat{B}$ in Equation (2) is estimated using the second-order polynomial, and its coefficients are contained in the navigation messages sent to receivers.

The corrected pseudorange $\rho_{c}$ in Equation (2) is a function of a number of unkowns:

(3) $$\rho_{c}^{i}\;=\;\sqrt{{\left(x^{i}\;-\;x_{u}\right)}^{2}\;+\;{\left(x^{i}\;-\;y_{u}\right)}^{2}\;+\;{\left(x^{i}\;-\;z_{u}\right)}^{2}}\;+\;b\;+\;\varepsilon_{u}^{i},$$

where i is the index for the satellite, i = 1,2…n, and n is the number of satellites; ($x_{u},\;y_{u},\;z_{u}$) denotes user position; ($x^{i}$, $y^{i}$, $z^{i}$) is the position of satellite i, computed from navigation messages; $\varepsilon_{u}^{i}$ is the residual error. In case of the single constellation GNSS, four measurements are required to determine the receiver position ($x_{u},\;y_{u},\;z_{u}$) and the receiver clock error b. If the number of the satellite constellations is s, then the number of unknowns is p = s + 3, and at least p measurements are required to solve Equation (3) for the unknowns. 

The iterative least squares method is a conventional method to solve for the unknowns [1]. The first step of the method is to derive the approximate pseudorange ${\hat{\rho }}_{c}^{i}$ based on the initial state: $x_{0}\;=\;({\hat{x}}_{u}$, ${\hat{y}}_{u}$, ${\hat{z}}_{u},\;\hat{b})$

(4) $${\hat{\rho }}_{c}^{i}\;=\;\sqrt{{\left(x^{i}\;-\;{\hat{x}}_{u}\right)}^{2}\;+\;{\left(y^{i}\;-\;{\hat{y}}_{u}\right)}^{2}\;+\;{\left(z^{i}\;-\;{\hat{z}}_{u}\right)}^{2}}\;+\;\hat{b}.$$

Next, the corrected pseudorange $\rho_{c}$ is linearized at the initial state

(5) $$\rho_{c}^{i}\;=\;{\hat{\rho }}_{c}^{i}\;+\;g^{i}∆x_{s}\;+\;\varepsilon^{i},$$

where $g^{i}$ represents the line of sight vector to the satellite i; $∆x_{s}$ is a vector of p unknown parameters. Equation (5) can also be represented as follows:

(6) $$∆\rho^{i}\;=\;g^{i}∆x_{s}\;+\;\varepsilon^{i},$$

where $∆\rho^{i}$ is the difference between the corrected pseudorange measurement and the estimated pseudorange:

$$∆\rho^{i}\;=\;\rho_{c}^{i}\;-\;{\hat{\rho }}_{c}^{i}.$$

Having n pseudorange measurements, the linearization Equation (6) can be written in the matrix form:

(7) $$∆\rho \;=\;G∆x_{s}\;+\;\varepsilon ,$$

where G is a n × p design matrix; ε is the vector of measurement errors. The least-squares solution of Equation (7) is given by

(8) $$∆x_{s}\;=\;{\left(G^{T}WG\right)}^{-1}G^{T}W∆\rho \;=\;{\left(G^{T}WG\right)}^{-1}G^{T}W\left(\rho \;-\;\hat{I}\;-\;\hat{T}\;+\;\hat{B}\;-\;{\hat{\rho }}_{c}\right),$$

where T represents the transpose operation of a matrix; W is the weight matrix. 

The user position and receiver clock are then updated based on the following equation:

(9) $$x_{s}\;=\;x_{0}\;+\;∆x_{s},$$

where the subscript s denotes the standalone GNSS. The magnitude of vector $∆x_{s}$ is compared with an acceptable value ξ to determine if the iteration aborts:

(10) $$\left|∆x_{s}\right|\;<\;\xi ,$$

where ξ is a small value that determines if the iteration stops. If the requirement in Equation (10) is satisfied, $x_{s}$ is regarded as the final solution. Otherwise, $x_{s}$ is then assigned to $x_{0}$, and the iteration continues from Equation (4) to Equation (9), until the requirement in Equation (10) is satisfied.

### 2.2. DGNSS in the Range Domain

The range domain DGNSS provides users with pseudorange corrections based on the reference station located at a known location $\left(x_{m},\;y_{m},\;z_{m}\right)$. The range between the reference station and the satellite i can be computed as

(11) $$R_{m}^{i}\;=\;\sqrt{{\left(x_{m}\;-\;x^{i}\right)}^{2}\;+\;{\left(y_{m}\;-\;y^{i}\right)}^{2}\;+\;{\left(z_{m}\;-\;z^{i}\right)}^{2}}.$$

Subtracting the pseudorange $\rho_{m}^{i}$ from the calculated range $R_{m}^{i}$ yields the pseudorange correction (PRC)

(12) $$PRC^{i}\;=\;R_{m}^{i}\;-\;\rho_{m}^{i}.$$

In the user receiver, the correction $PRC^{i}$ is then employed to reduce various errors

(13) $$\rho_{c}^{i}\;=\;\rho^{i}\;+\;PRC^{i},$$

where $\rho_{c}^{i}$ is the corrected pseudorange. Using the corrected pseudorange, one can calculate the position using the iterative least squares method discussed in the previous subsection.

Because the satellite clock error is the same for the reference station and the user, the above differencing process can remove all of the satellite clock errors. Moreover, because the propagation errors and the orbit errors are correlated over space, most errors due to the satellite orbit and the propagation delay can be reduced significantly. The corrected pseudorange is then used to determine user position. This process is usually achieved based on the iterative method presented in the previous subsection.

### 2.3. NMEA Standard

As discussed previously, the GNSS chipsets utilize the pseudorange measurements to determine the user location. The results, e.g., the positioning information and the satellite information, are usually delivered to users through the NMEA 0183 protocol, which is supported by all chipsets for exchanging the processed measurements.

Android smartphones employ NMEA messages to deliver the positioning results. Several NMEA messages are supported by these smartphones, such as the fix data (GGA), the recommended minimum data (RMC), the overall satellite data (GSA), and the detailed satellite data (GSV) messages. In particular, the DGNSS infrastructure presented in the next section utilizes two of these messages: the GGA message and the GSA message. The GGA message gives the position fix in WGS-84, while the GSA message delivers a list of satellites that contribute to determining the position fix. As an example, the definition of the GSA message is explained in Table 1. One GSA message can contain up to 12 active satellites. There can be several GSA messages for one epoch when more than one constellation is used.

## 3. DGNSS Positioning Infrastructure

The fundamentals of DGNSS based on NMEA messages are presented in this section. A positioning infrastructure is then proposed and described, with the emphasis on the architecture and the implementation issues.

### 3.1. DGNSS Based on NMEA Messages

Android smartphones provide the standalone position ($x_{0}\;=\;\left(x_{s},\;y_{s},\;z_{s}\right)$) via the NMEA GGA message. More importantly, the NMEA GSA message provides all satellite IDs that contribute to the standalone position. The DGNSS based on NMEA messages starts from standalone position $x_{0}\;=\;\left(x_{s},\;y_{s},\;z_{s}\right)$, and attempts find the position correction $∆x_{d}$, such that

(14) $$x_{d}\;=\;x_{0}\;+\;∆x_{d},$$

where $x_{d}$ is the solution from the range domain DGNSS. In this subsection, we will derive $∆x_{d}$ using the range domain DGNSS first, and then attempt to represent $∆x_{d}$ with the known information.

As the first step of the iterative least squares method, the approximate range between the satellite and the user can be estimated based on the standalone user position $x_{0}\;=\;\left(x_{s},\;y_{s},\;z_{s}\right)$:

(15) $${\hat{\rho }}_{c}^{i}\;=\;\sqrt{{\left(x_{s}\;-\;x^{i}\right)}^{2}\;+\;{\left(y_{s}\;-\;y^{i}\right)}^{2}\;+\;{\left(z_{s}\;-\;z^{i}\right)}^{2}}\;+\;{\hat{b}}_{u}.$$

If we know the pseudorange $\rho^{i}$, then the observable $∆\rho^{i}$ is obtained as

(16) $$∆\rho^{i}\;=\;\left(\rho^{i}\;+\;PRC^{i}\right)\;-\;{\hat{\rho }}_{c}^{i}.$$

If more than p satellites are tracked, then the least-squares position correction $∆x_{d}$ can be calculated, as done in (8)

(17) $$∆x_{d}\;=\;{\left(G^{T}WG\right)}^{-1}G^{T}W∆\rho =\;{\left(G^{T}WG\right)}^{-1}G^{T}W\left(\rho \;+\;PRC\;-\;{\hat{\rho }}_{c}\right),$$

where G is the design matrix, which can be calculated from the initial user position and satellite positions; ρ is the pseudorange measurement, which is not available from the perspective of developers; PRC represents the pseudorange corrections from the reference station.

As shown in Equation (17), the range-domain DGNSS calculates $∆x_{d}$ based on the pseudorange measurements, which are not available to Android smartphone developers. However, we can rewrite Equation (17) as follows:

(18) $$∆x_{d}\;=\;{\left(G^{T}WG\right)}^{-1}G^{T}W\left[\left(\rho \;-\;\hat{I}\;-\;\hat{T}\;+\;\hat{B}\;-\;{\hat{\rho }}_{c}\right)\;+\;\left(\hat{I}\;+\;\hat{T}\;-\;\hat{B}\;+\;PRC\right)\right],$$

where $\hat{I}$, $\hat{T}$, and $\hat{B}$ are estimates of the ionospheric delay, the tropospheric delay, and the satellite clock error with the broadcast messages, respectively. Substituting (7) into (18) gives

(19) $$∆x_{d}\;=\;∆x_{s}\;+\;{\left(G^{T}WG\right)}^{-1}G^{T}W\left(\hat{I}\;+\;\hat{T}\;-\;\hat{B}\;+\;PRC\right)$$

because $∆x_{s}$ is a very small value (shown in Equation (10)), the position shift $∆x_{d}$ can be approximated as follows:

(20) $$∆x_{d}\;\approx \;{\left(G^{T}WG\right)}^{-1}G^{T}W\left(\hat{I}\;+\hat{\;T}\;-\;\hat{B}\;+\;PRC\right)$$

It can be seen from Equation (20) that the position shift can be calculated even if the pseudorange measurements are not provided.

The position correction in Equation (20) is determined by the following vectors: G, $\hat{I}$, $\hat{B,}$ and $\hat{T}$, as well as PRC. The distance from the satellite and the receiver can be as large as 20,000 km., and the accuracy of the satellite position and the receiver position required has limited effects on the positioning results, especially for the meter level positioning. Therefore, the geometry matrix G can be calculated from navigation messages, and it can also computed using the GSV sentences [17,20]. The propagation delays $\hat{I}$ and $\hat{T}$ are computed with the standard models. The satellite clock error is estimated using the navigation messages.

**Figure 1:**
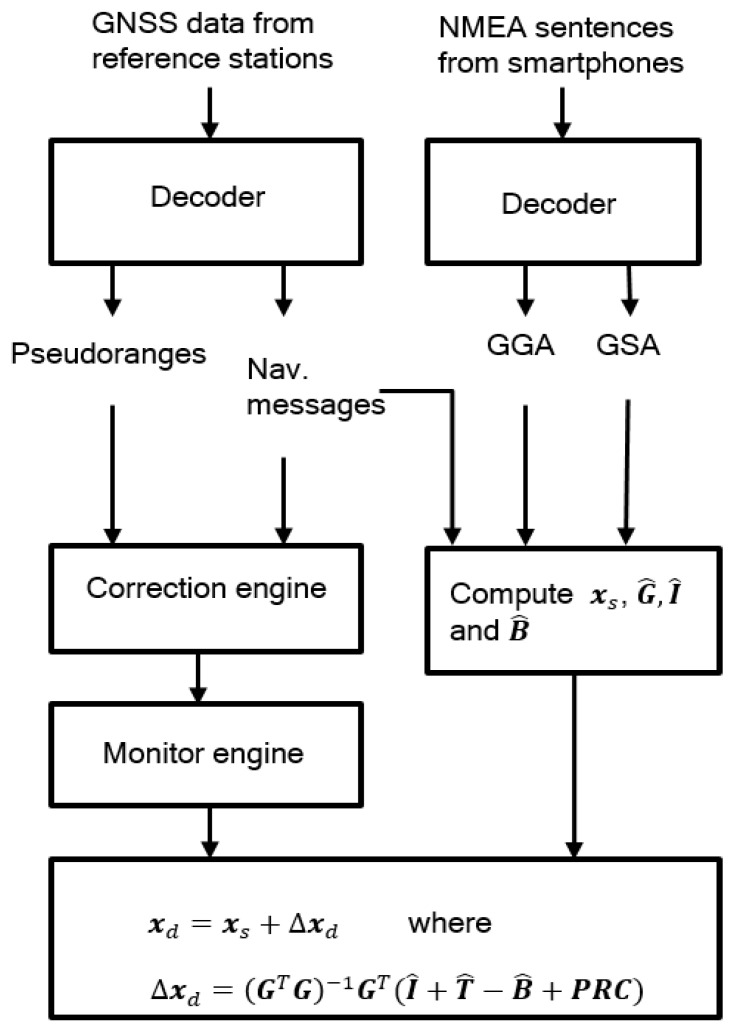
Flowchart of Differential Global Navigation Satellite System (DGNSS) based on NMEA messages.

The position correction is valid when these vectors are based on the set of satellites, which are utilized in determining the standalone GNSS position. As shown in Figure 1, a critical step of DGNSS based on NMEA messages is that, for each satellite ID that appears in GSA sentences, we compute the propagation delays, the pseudorange correction, and the estimate of the satellite clock error based on the data from the reference station. The above vectors are then formed on the order of satellites that appear in the GSA sentence.

### 3.2. Assumptions on DGNSS Based on NMEA Messages

The DGNSS based on NMEA messages works on some essential assumptions regarding the GNSS chipsets embedded in smartphones:

- The GNSS chipsets apply the standard Klobuchar model to estimate the ionospehric delay.
- There are some other methods to solve for unknowns, such as the closed-form method and the Kalman filtering method. The proposed DGNSS assumes that the conventional iterative least squares method is applied.
- The satellite measurements are weighted based on the satellite elevation.

The performance of the proposed DGNSS is thus dependent on the model and the algorithms used in the chipsets. In the proposed DGNSS, it is assumed that the most conventional model is applied. This also indicates that the direct cooperation with chipset manufacturers is required in the future to understand the method and the model used in the chipsets.

### 3.3. Implementation of the Infrastructure

**Figure 2:**
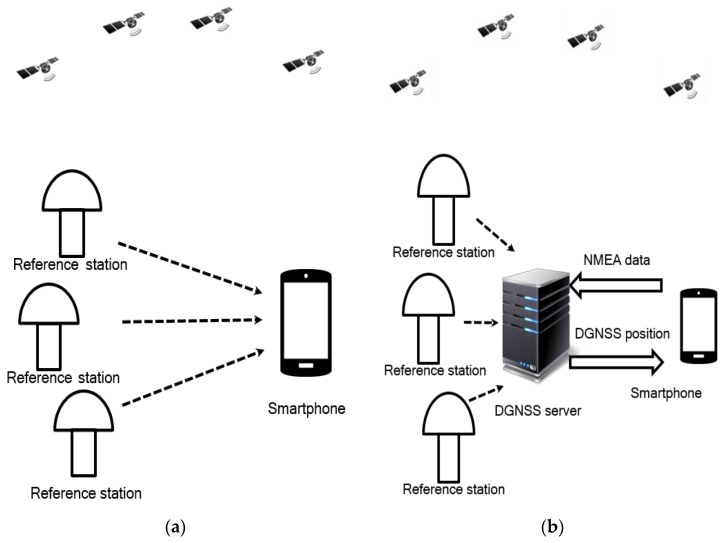
(**a**) use of a smartphone to calculate the DGNSS positions; (**b**) use of a server to calculate the DGNSS positions.

There are two approaches to implementing the DGNSS based on NMEA messages. One approach is to transmit directly the reference station data to smartphones, which is in turn integrated with the NMEA messages in the smartphones. The smartphone performs most of the calculations. Figure 2a illustrates this approach. It consists of some reference stations that provides the GNSS data to a smartphone in a specific protocol, e.g., Ntrip. In the smartphone, the reference station data and the NMEA messages are processed in the manner as specified in Figure 1. In this simple method, the reference station data are transmitted from the reference station directly, and only one-way communication is required. However, because of the limited computational capacities of smartphones, it is challenging to implement the DGNSS algorithm in the smartphone, including the range correction engine, the monitor engine, and the position engine. For example, the position correction calculation in (20) requires the multiplication and inversion operation of the matrix, and the matrix can have tens of rows in the case of multiple satellite constellations. Such a calculation can be challenging to perform using a smartphone.

The other approach is to use a server collect GNSS data from reference stations, as shown in Figure 2b. When the smartphone requests the DGNSS service, it transmits the NMEA messages to the server. For each request, the server computes the DGNSS position using the NMEA messages from the smartphone. The DGNSS position is then transmitted to the smartphone. In this method, the server performs most of the calculations in the architecture. The smartphone in this method must transmit the NMEA messages to the server and must receive the DGNSS position from the server. Therefore, two-way communication is required for the smartphone. The first advantage of this method is that most of the calculations are performed by the DGNSS server efficiently, i.e., the smartphone does not have to consider these calculations. The second advantage is that the range correction engine and the monitor engine are common parts for all smartphones that request the DGNSS service. These two engines can be performed simultaneously at the server, and, in turn, the range corrections are utilized by all smartphones that are connected to the server.

To reduce the user’s computational requirement, we developed the DGNSS infrastructure based on the second method. The infrastructure consists of a server program responsible for providing DGNSS service, and a Software Development Kit (SDK) capable of sending the NMEA messages to the server and receiving the DGNSS positions from the server. Both programs can be configured with different settings, such as the IP address and the port number.

## 4. Experimental Studies

**Figure 3:**
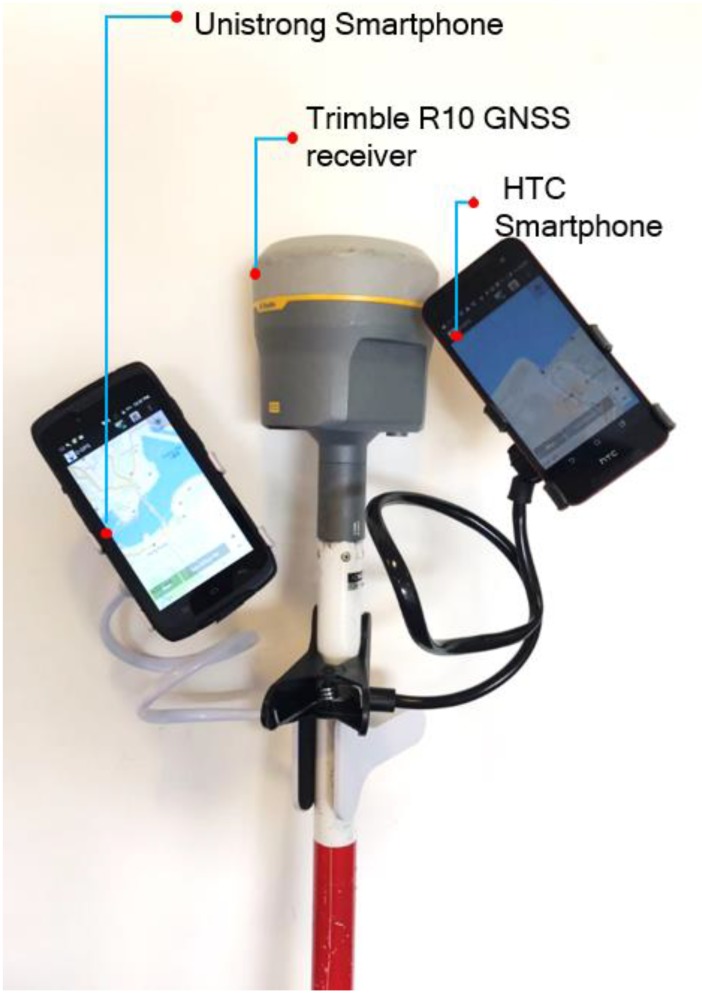
Experimental system.

Extensive tests have been conducted to evaluate the performance of the proposed infrastructure. In each test, the standalone and DGNSS positions from smartphones are compared with the ground truth from a surveying-grade receiver. The Trimble GNSS receiver in Figure 3 receives RTK corrections from reference stations, and then solves for integer ambiguities to determine positions up to a few centimeters. Because smartphones offer the meter-level accuracy, this Trimble receiver is sufficient for evaluating the performance of the DGNSS based on NMEA messages.

As shown in Figure 3, the DGNSS App was installed in two smartphones, a Unistrong smartphone from Beijing, China, and an HTC smartphone from Daejeon, Korea. The model of the Unistrong smartphone is A3S, and it integrates a NEO-7M GNSS module manufactured by the U-Blox company in Thalwil, Switzerland. This NEO-7M supports GPS and Beidou satellite systems. The version of the operating system of the Unistrong smartphone is Android 7.0.1. We also use the HTC One smartphone in the test, and it uses the Android operating system version 4.1. The CPU model of the HTC smartphone is Qualcomm Adreno 320. The satellite systems supported by HTC One include GPS and Glonass. The DGNSS App running in both smartphones is responsible for data communication and displaying the real-time positions on a map. The app also records the standalone and DGNSS positions in the log files for further analysis.

The devices (the Trimble receiver and two smartphones) record positions in log files at the sampling rate of 1 Hz. For each test, the log files contain three types of positions: standalone and DGNSS solutions from smartphones, and RTK solutions from the Trimble receiver. For each epoch, the positions of the standalone GNSS and DGNSS from smartphones are compared with the position from the Trimble receiver, and the positioning errors are calculated.

Some representative tests are presented in the following three subsections. The walking test is shown in the next subsection. Next, the walking tests in the second subsection show the effects of the ionosphere on the smartphone positioning in low-latitude areas and show how the infrastructure can mitigate them. Finally, the performance of the infrastructure in urban areas is illustrated.

### 4.1. Walking in the Open Area

**Figure 4:**
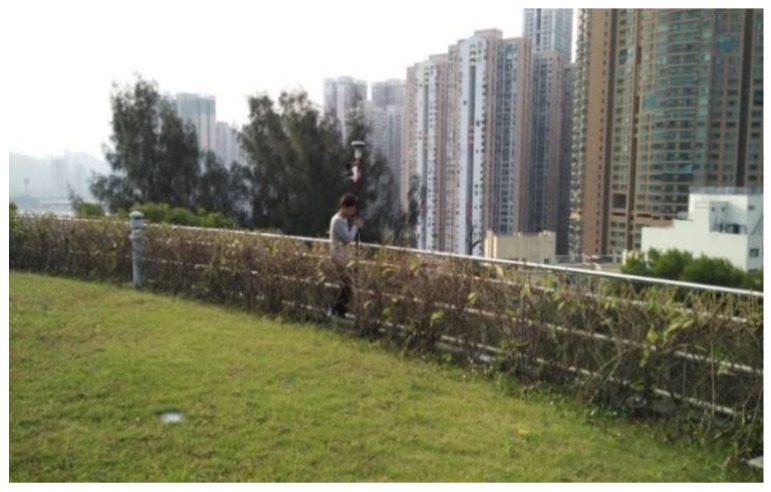
Experiment on an observation deck in Macau, China.

The open area test was performed to evaluate the proposed infrastructure on a circular observation deck in Macau, China, as shown in Figure 4. The radius of the circular deck is approximately 30 m. The distance between the smartphones and the reference station is approximately 6 km. There are some buildings approximately 1 km away. As shown in the figure, a pedestrian holding the pole walked along the specific tracks from 3:00 p.m. to 4:00 p.m. on 30 November 2016.

**Figure 5:**
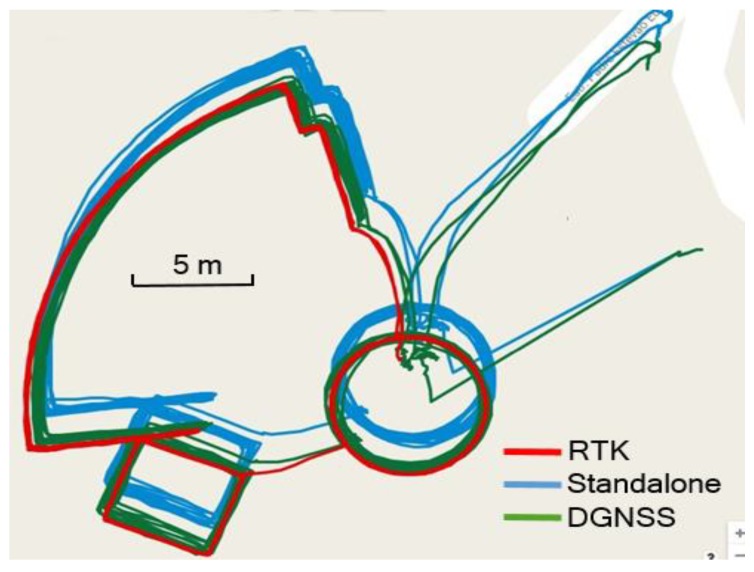
Positioning results in the open area, the trajectories of real-time kinematic positioning (RTK) receiver (red line), GNSS (blue line) and DGNSS (green line) from the Unistrong smartphone.

Figure 5 shows the trajectories using different methods. As shown, the pedestrian walked along three pre-defined routes: a portion of a circle sector, a small circle, and a rectangle. In the figure, the red trajectory represents the ground truth, and the blue and green trajectories represent GNSS and DGNSS solutions in the smartphone, respectively. There was a significant bias between the standalone GNSS solution and the ground truth. Figure 5 shows that most of the system errors were removed using the proposed infrastructure.

We also compared the positioning error of GNSS with that of DGNSS in the smartphone. Table 2 shows that the horizontal average positioning error is 2.3 m and the RMS error is 2.6 m when using standalone GNSS in the smartphone. With the proposed DGNSS, the horizontal average positioning error is reduced to 0.8 m and the RMS error is reduced to 1.5 m.

**Figure 6:**
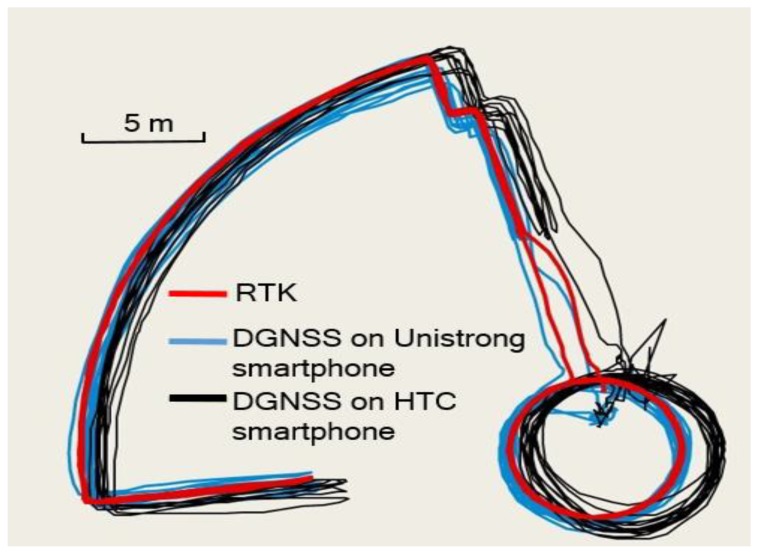
Performance comparison between two smartphones.

The performance of the proposed infrastructure was also assessed in different types of smartphones. As shown in Figure 6, the DGNSS trajectory of the Unistrong smartphone is shown as the blue line, while that of HTC is shown as a black line. The positioning accuracy is found to be improved in both smartphones. The DGNSS performance in the Unistrong smartphone is better than that on the HTC smartphone. The difference can be caused by a number of factors, such as different algorithms and models employed in smartphone GNSS chipsets.

### 4.2. Effects of Ionosphere in the Low-Latitude Region

It is well known that the ionospheric activity is affected by many factors, such as daytime, season, and solar activity. Thus, these factors may affect the positioning accuracy of the standalone GNSS. When using the DGNSS positioning infrastructure, most of the ionospehric errors can be mitigated. In this paper, we mainly compared the performance of DGNSS in the morning and that in the afternoon.

Macau is located in low-latitude regions, with the latitude ranging from 22.1 degrees to 22.3 degrees. A comparison test was performed in Macau on 8 December 2016, when the solar activity was low. The first data set was collected from 10:30 a.m. to 11:00 a.m., during which the ionospheric delay was still small. The other was collected from 2:00 p.m. to 2:30 p.m., during which the ionospheric delay reached the diurnal maximum [10]. In two tests, we walked along a predefined track of a trapezoid shape. Both trajectories are in the open area, and the effects of multipath on smartphones can be assumed to be same for two data sets. The distance between the smartphones to the reference station is approximately 4 km.

**Figure 7:**
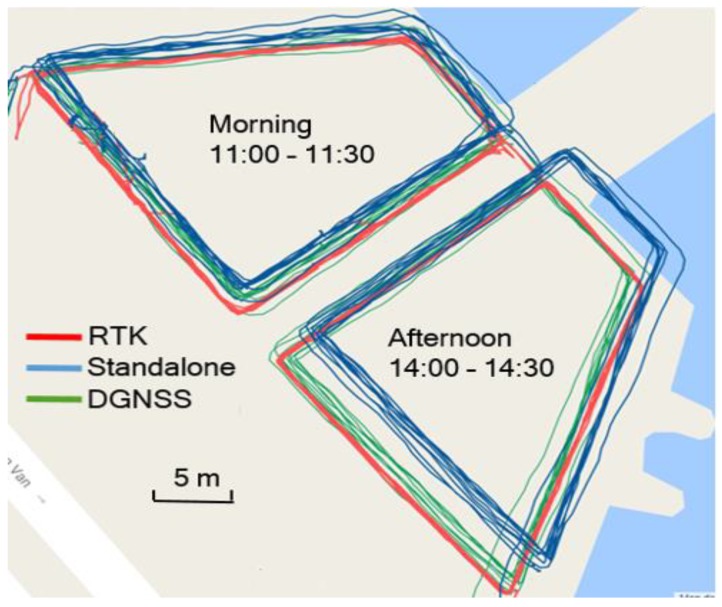
Smartphone performance comparison between the morning and the afternoon.

Figure 7 compares the smartphone positioning performance in the morning and that in the afternoon. In the figure, the blue line and the green line represent the smartphone GNSS and DGNSS trajectories respectively, and the red line represents the ground truth. As shown in the figure, the standalone GNSS positioning error in the morning was much smaller than that in the afternoon. When using the positioning infrastructure, the positioning error was reduced significantly for both tests.

Table 3 shows the smartphone performance in the morning and that in the afternoon. In the morning, the ionospheric delay was smaller, and the average of the standalone GNSS positioning error was 0.9 m, and the RMS error was 1.7 m. In the afternoon, the ionospheric delay was much larger; the performance of the traditional model used in smartphones was not good, especially for low latitude areas. During this period of time, the average of the standalone GNSS positioning error reached 4.0 m, and the RMS error was as large as 4.4 m. When using the proposed DGNSS, the positioning error was reduced significantly in both tests. Specifically, the RMS error was reduced to 1.5 m, and 1.8 m in the morning and the afternoon, respectively. The average of horizontal positioning error was reduced to 0.4 m in both tests.

### 4.3. Walking in Urban Areas

**Figure 8:**
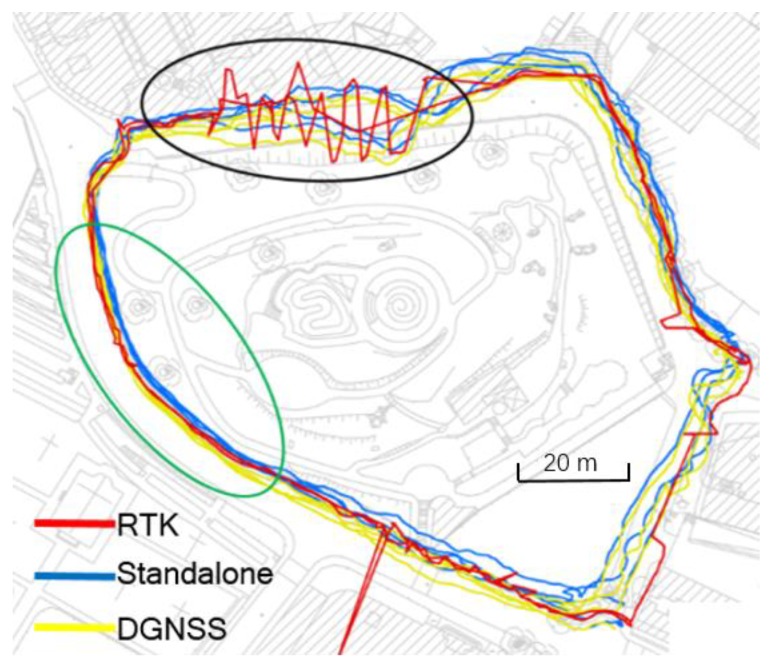
Trajectory of the pedestrian in the urban area.

Another test was also conducted to evaluate the positioning performance of smartphones in the urban area. In the test, the pedestrian holding the equipment walked around a mountain. The distance between the reference station and the mountain is approximately 2.5 km. Figure 8 shows the trajectory of the pedestrian. Along the sidewalks shown in the green oval, the GNSS signals from satellites were affected by the trees, and the multipath error was not large. The accuracy improvement by using the infrastructure can still be seen from the figure. When the pedestrian was in the section denoted by the black oval, however, the multipath error was very large, and the Trimble receiver could not fix the ambiguities. In this section, the positioning accuracy of smartphones was not improved significantly. This result agrees with the fact that the differencing process could not remove multipath errors. The mitigation of the multipath errors on the GNSS positioning will be conducted in our future study.

## 5. Conclusions

This paper described the DGNSS positioning infrastructure that delivers improved accuracy to a huge number of users. The infrastructure enables most of calculations to be performed in the server side, bringing benefits to all of relevant smartphones. When implementing the DGNSS infrastructure, all smartphone types were considered. Because different satellite systems are supported by different smartphones, the infrastructure developed in this paper considers all the combinations of satellite systems used in smartphones.

A comparison of the proposed infrastructure against the ground truth, for all tests performed in open areas, demonstrated that the RMS horizontal positioning error was reduced to less than 2 m. The test in the open area showed that average horizontal positioning errors were reduced from 2.3 m to 0.8 m, and the RMS horizontal error was reduced from 2.6 m to 1.5 m. The strength of DGNSS is particularly apparent in low-latitude or high-latitude regions, where it removes most of the errors caused by the ionosphere. Another test in Macau (low-latitude regions) showed that the positioning error of the standalone smartphone in the afternoon was much larger than that in the morning. Although the test was performed in 2017 when the solar activity reached almost the minimum, the average horizontal positioning error reached 4.0 m, and the RMS error was as large as 4.4 m. With the infrastructure, the average horizontal positioning error was reduced to 0.4 m, and the RMS error was reduced to 1.8 m.

It is well known that the DGNSS operation does not reduce the receiver related error (e.g., multipath errors). The receiver related error must be considered by DGNSS in urban areas especially for safety-critical applications, for example, the lane-level positioning system and the collision warning system. In this study, the performance of DGNSS in urban areas was studied, and the test demonstrated that the DGNSS still adds value in the urban environments.

We also compared the DGNSS based on NMEA messages with the range-domain DGNSS; the comparison showed that the accuracy of a smartphone with infrastructure is still worse than that of range-domain DGNSS on surveying grade devices. Based on the results, the RMS error of range-domain DGNSS was less than 1 m, and the accuracy of the smartphone positioning with the infrastructure was approximately 1.5 m. The performance of the infrastructure depends on a number of factors, such as the model applied in the chipsets, the position determination algorithms and the weighing methods, as well as the performance of the antenna. In the current infrastructure, we assumed that the most conventional models and algorithms are used in the GNSS chipsets. To obtain the higher accuracy in the future, the direct cooperation with chipset manufactures is required to understand the models and algorithms used in the chipsets.

## Figures and Tables

**Table 1:** GSA sentence content.

Position	Content and Explanation
1	Header of GSA (the prefixes GP, GL, and BD represent GPS, Glonass and Beidou satellites, respectively)
2	Mode (2D, 3D or fix not available)
3–14	Satellite IDs for the position fix
15–17	Dilution of Precision (DOP) values

**Table 2:** Performance comparison between standalone GNSS and DGNSS in the smartphone.

Mode	Average/m	Root Mean Squares (RMS)/m
Standalone GNSS	2.3	2.6
DGNSS	0.8	1.5

**Table 3:** Smartphone performance in the morning and in the afternoon.

Test	Mode	Average	RMS
Morning	Standalone	0.9	1.7
	DGNSS	0.4	1.5
Afternoon	Standalone	4.0	4.4
	DGNSS	0.4	1.8
